# Supplementary material for: LUBAC is required for RIG-I sensing of RNA viruses
Source: Cell Death Differ. 2023 Nov 24;31(1):28–39. doi: 10.1038/s41418-023-01233-x (PMC10781740; doi:10.1038/s41418-023-01233-x)
Supplement: Supplementary file 2 — Supplementary data [file 41418_2023_1233_MOESM2_ESM.pdf]

Supplementary data

Supplementary Table 1. qPCR primer sequences used in this study.

Gene symbol	Gene name	Primer direction	Primer sequence
GAPDH	glyceraldehyde-3-phosphate dehydrogenase	Forward	ACC CAG AAG ACT GTG GAT GG
		Reverse	TTC TAG ACG GCA GGT CAG GT
IFNB1	interferon beta 1	Forward	ACA TCC CTG AGG AGA TTA AGC A
		Reverse	GCC AGG AGG TTC TCA ACA ATA G
CXCL10	C-X-C motif chemokine ligand 10	Forward	GTG GCA TTC AAG GAG TAC CTC
		Reverse	GCC TTC GAT TCT GGA TTC AGA CA
IFNL1	interferon lambda 1	Forward	CGC CTT GGA AGA GTC ACT CA
		Reverse	GAA GCC TCA GGT CCC AAT TC
ISG15	ISG15 ubiquitin like modifier	Forward	AGC ATC TTC ACC GTC AGG TC
		Reverse	GAG GCA GCG AAC TCA TCT TT
ISG54/IFIT2	interferon induced protein with tetratricopeptide repeats 2	Forward	CTG AAG AGT GCA GCT GCC TG
		Reverse	CAC TTT AAC CGT GTC CAC CC
NFKBIA	NFKB inhibitor alpha	Forward	CTC CGA GAC TTT CGA GGA AAT
		Reverse	GCC ATT GTA GTT GGT AGC CTT
IL6	interleukin 6	Forward	ACA ACC ACG GCC TTC CCT ACT T
		Reverse	CAC GAT TTC CCA GAG AAC ATG TG

Human qPCR primer sequences

Gene symbol	Gene name	Primer direction	Primer sequence
Hprt	hypoxanthine guanine phosphoribosyl transferase	Forwards	GTT GGA TAC AGG CCA GAC TTT GTT G
		Reverse	GAT TCA ACT TGC GCT CAT CTT AGG C
Ifnb1	interferon beta 1	Forwards	GCC TAG GTG AGG TTG ATC T
		Reverse	AGC TCC AAG AAA GCA CGA ACA T
Cxcl10	C-X-C motif chemokine ligand 10	Forwards	ACT GCA TCC ATA TCG ATG AC
		Reverse	TTC ATC GTG GCA ATG ATC TC
Isg56/Ifit1	interferon-induced protein with tetratricopeptide repeats 1	Forwards	CTG AAG AGT GCA GCT GCC TG
		Reverse	CAC TTT AAC CGT GTC CAC CC

Isg15	ISG15 ubiquitin like modifier	Forwards	GCA AGC AGC CAG AAG CAG ACT CC
		Reverse	CGG ACA CCA GGA AAT CGT TAC CCC
Il6	interleukin 6	Forwards	GTA GCT ATG GTA CTC CAG AAG AC
		Reverse	ACG ATG ATG CAC TTG CAG AA
Nfkbia	NFKB inhibitor alpha	Forwards	CTG CAG GCC ACC AAC TAC AA
		Reverse	CAG CAC CCA AAG TCA CCA AGT

Murine qPCR primer sequences

Supplementary Table 2. Primary and secondary antibodies used for immunoblotting in this study.

Antibody	Company	Code	Dilution/diluent
RIG-I (D-12)	Santa Cruz	sc-376845	1:1000/TBST
MAVS (E-3)	Santa Cruz	sc-166583	1:1000/TBST
IKKgamma/NEMO (DA10-12)	Cell Signaling Technology	#2695	1:1000/TBST
IRF3 [EPR2418Y]	Abcam	ab68481	1:1000/TBST
NAK/TBK1 [EP611Y]	Abcam	ab40676	1:1000/TBST
IκBα (L35a5)- MEF	Cell Signaling Technology	#4814	1:1000/TBST
α-Tubulin (DM1A)	Millipore	05-829	1:5000/TBST
ZIKV E protein	GeneTex	GTX133314	1:1000 PBST
GAPDH	Sigma	G8795	1:20000 PBST
IRF3 (phospho S386) [EPR2346]	Abcam	ab76493	1:1000/TBST
Phospho-TBK1 (Ser172) D52C2	Cell Signaling Technology	#5483S	1:1000/TBST
Phospho-IκBα (Ser32/36) (5A5)	Cell Signaling Technology	#9246	1:1000/TBST
Phospho-IRF3 (Ser396) (4D4G)	Cell Signaling Technology	#4947	1:500/TBST
Ku70 [N3H10]	Abcam	ab3114	1:1000/TBST
IKKε (D61F9) XP	Cell Signaling Technology	#3416	1:500/TBST
HOIP (human; full length), pAb	Ubiquigent	68-0013-100	1:1000/TBST
RBCK1 (H-1) (HOIL-1)	Santa Cruz	sc-393754	1:1000/TBST
SHARPIN	ProteinTech	14626-1-AP	1:1000/TBST
Flag	Sigma	#F7425	1:1000/TBST

Primary antibodies used for western blotting

**Antibody**	**Company**	**Code**	**Dilution/diluent**
Goat anti-rabbit 680 RD	Li-Cor	926-68071	1:10000/TBST
Goat anti-mouse 800 CW	Li-Cor	926-32210	1:10000/TBST
Donkey anti-Goat 800-CW	Li-Cor	926-32214	1:10000/TBST

Secondary antibodies used for western blotting

Supplementary Table 3. Antibodies used for PhosFlow analysis in this study

Antibody	Company	Code	Dilution/diluent
Phospho-IRF-3 (Ser396) (D6O1M) Rabbit mAb (Alexa Fluor® 647 Conjugate)	Cell Signaling	#10327	1:25/PBS 1% FCS
PE Rabbit Anti- Active Caspase-3 Clone C92-605	BD Pharmingen	550821	1:10/PBS 1% FCS

Antibodies used for phos-flow analysis

Supplementary Table 4. Antibodies used for immunofluorescence analysis in this study

Antibody	Company	Code	Dilution
IRF-3 (D83B9) Rabbit mAb	Cell Signaling	4302	1:200
NF-κB p65 (C-20)	Santa Cruz	312	1:100
E-Protein 4G2	Fiocruz-PR, Brazil	-	1:100
Human monoclonal antibody DV 18.4	Beltramello *et al*. 2010	-	1:100

Primary antibodies used for immunofluorescence

Antibody	Company	Code	Dilution
Goat anti-Rabbit IgG (H+L) Alexa Fluor 568 conjugated	Invitrogen	A-11011	1:2000
Goat anti-Mouse IgG (H+L) Alexa Fluor 488 conjugated	Invitrogen	A-11001	1:2000
Rabbit anti-Mouse IgG (H+L) Alexa Fluor 568 conjugated	Invitrogen	A-11061	1:2000
Goat anti-Human IgG (H+L) Alexa Fluor 488 conjugated	Invitrogen	A-11013	1:2000

Secondary antibodies used for immunofluorescence

Supplementary Figure S1

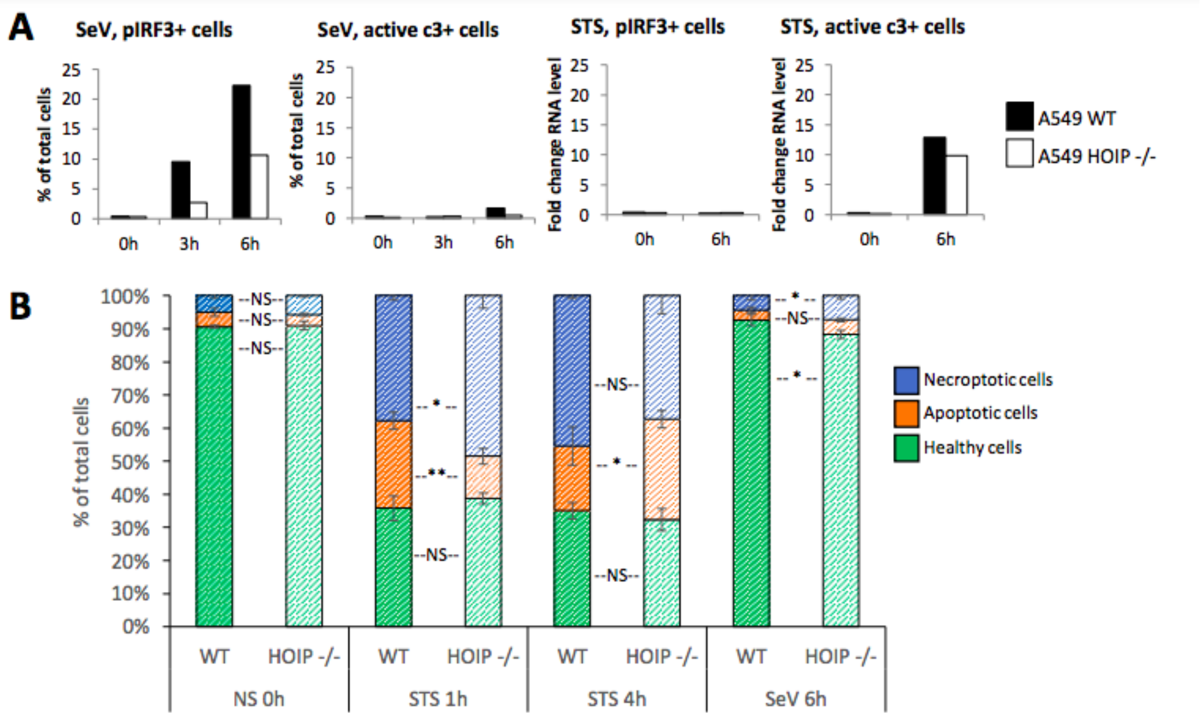

Supplementary Figure S1: **Loss of HOIP does not result in RIG-I-driven cell death in A549 cells** A) Phos-flow to measure cells expressing phospho-IRF3 and cleaved caspase 3 in A549 WT and HOIP -/- cells infected with SeVat 1:300 dilution. B) Nucleocounter (NC-250) Vitality Assay in A549 WT and HOIP -/- cells treated with staurosporine at 2 µM or infected with SeV at 1:300 dilution for the indicated times. NS = not stimulated.

Supplementary Figure S2

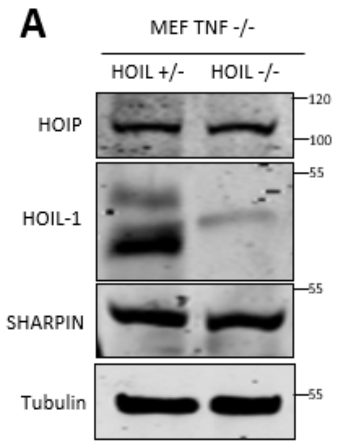

Supplementary Figure S2: Western blotting analysis of LUBAC components in MEF TNF -/- HOIL +/- and TNF -/- HOIL -/- cells.

Supplementary Figure S3

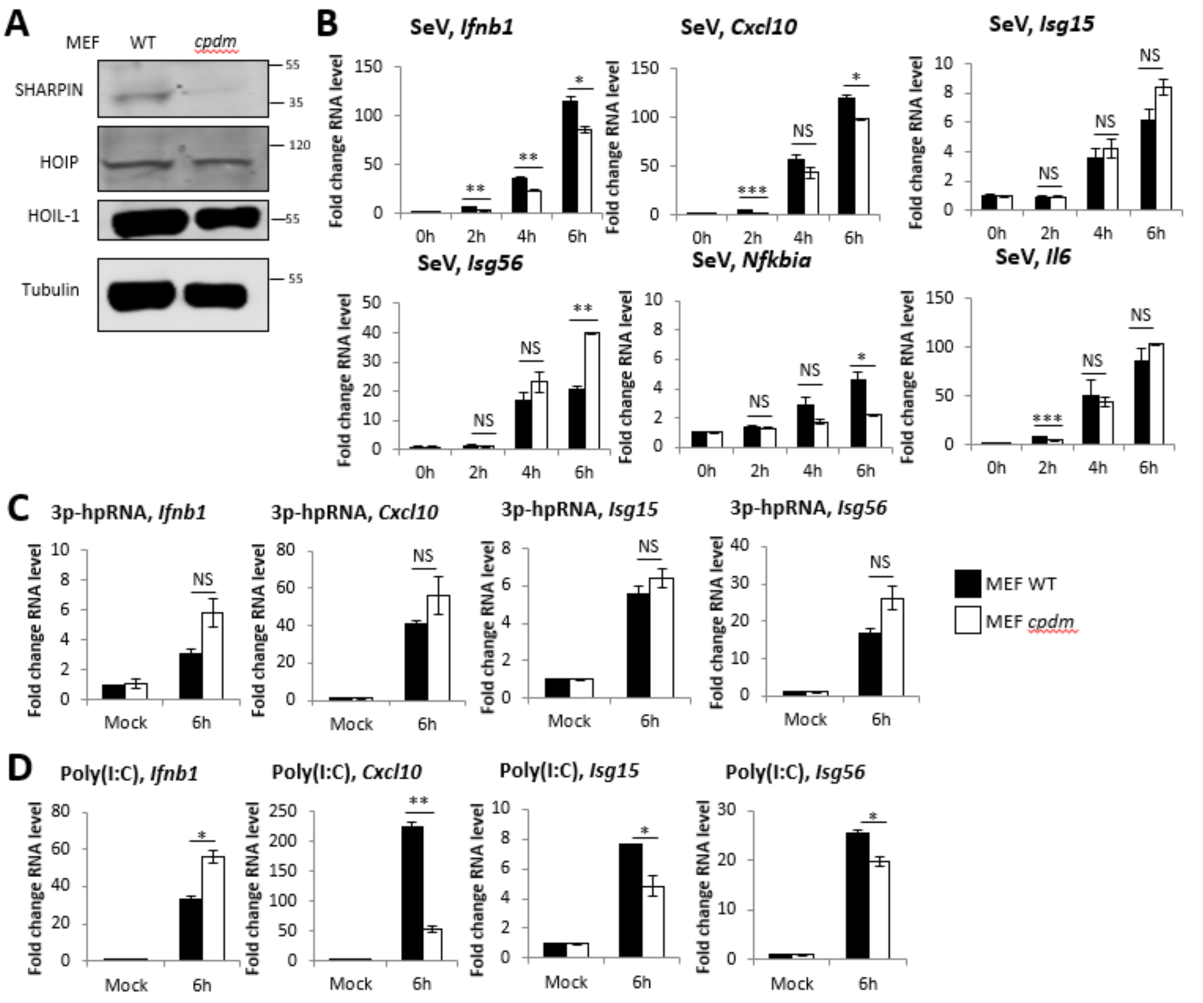

Supplementary Figure S3: SHARPIN is not required for RIG-I immune response to SeV and synthetic RNAs in MEF cells A) Western blotting analysis of MEF WT and *cpdm* cells. qPCR to measure transcription of indicated genes in MEF WT and *cpdm* cells B) infected with SeV at a 1:300 dilution or transfected with C) 1 μg 3p-hpRNA and D) 1 μg Poly(I:C).

Supplementary Figure S4

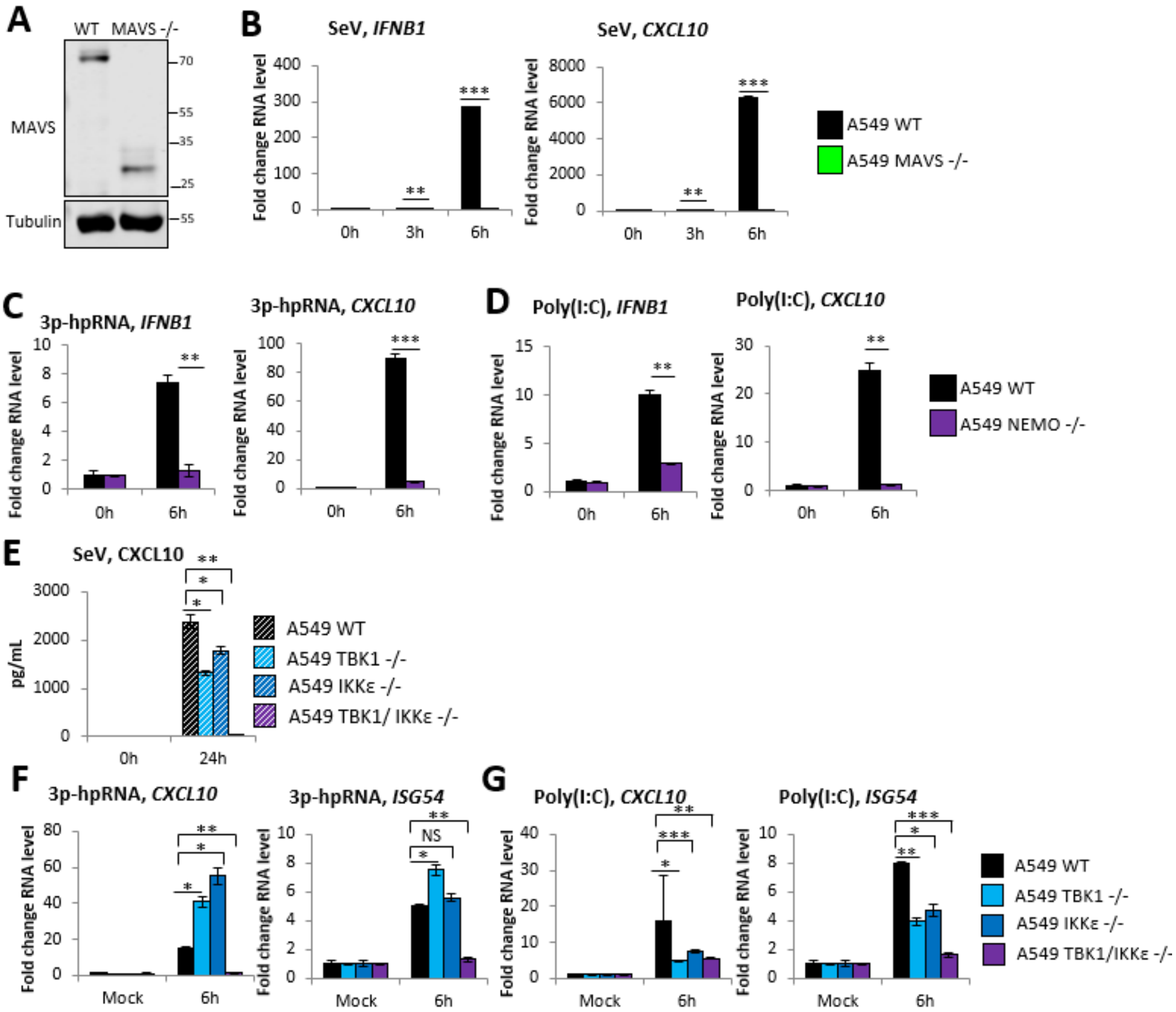

Supplementary Figure 4: Requirement of TBK1, IKKε, NEMO and MAVS in RIG-I signalling

A) Western blotting analysis of A549 WT and MAVS -/- cells. B) Transcription of indicated genes measured by qPCR in A549 WT and MAVS -/- cells infected with SeV at 1:300 dilution. qPCR to measure transcription of indicated genes in A549 WT and NEMO -/- cells transfected with C) 1 µg 3p-hpRNA and D) 1 µg Poly(I:C). A549 WT, TBK1 -/-, IKKε -/- and TBK1/IKKε -/- cells E) infected with SeV at 1:300 and ELISA to measure CXCL10 secretion, transfected with F) 1 µg 3p-hpRNA and G) 1 µg Poly(I:C) and qPCR to measure transcription of indicated genes.
